# The Intra-Muscular Method in the Treatment of Syphilis

**Published:** 1905-08-26

**Authors:** 


### THE INTRA-MUSCULAR METHOD IN THE TREATMENT OF SYPHILIS.

Lieutenant - Colonel Lambkin,[1] R.A.M.C., considers that the means at our disposal for the treatment of syphilis have in recent years vastly improved, but that full advantage has not been taken of them. Hence, he argues, though the immediate signs and symptoms of the disease have been ameliorated, the later effects in the shape of general paralysis of the insane, tabes dorsalis, etc., still show a terrible record. For this, the ineffective methods of treatment adopted in civil practice are, Colonel Lambkin holds, largely responsible. These methods formerly were also used in military practice, but they have now been replaced by systematic intra-muscular injections, with the result that the ratios per 1,000 of invalids sent home, and of those requiring to be discharged as permanently unfit, have been materially reduced. The preparation generally used in the army is standardised so that 10 minims contain 1 grain of metallic mercury. When the patient is discharged from hospital, the nature of his disease and the urgent necessity for continuous treatment are explained to him, and he is advised to present himself every week or fortnight, when he receives an injection equivalent to one grain of mercury. This is continued for a period of not less than three months. Afterwards the treatment is suspended for two months, during which time he is kept under observation, and if there has been no recurrence it is probable he is cured. It is found that the men willingly and even anxiously return for the weekly injection when they are once made to understand the necessity for it. Colonel Lambkin argues that the same plan is equally applicable in civil practice, and combats the opinion that the pain caused by the injection is a serious difficulty. In support of this he quotes the experience of Dr. Henry FitzGibbon, senior surgeon to the Dublin Lock Hospital. Medication by the mouth, he contends, is rarely practised by the patient in the regular and sustained manner necessary to obtain continuous action of the remedy and complete cure of the disease.

[1] Brit. Med. Jour., Aug. 19, 1905.

